# Transcriptome analysis of miRNA and mRNA in the livers of pigs with highly diverged backfat thickness

**DOI:** 10.1038/s41598-019-53377-x

**Published:** 2019-11-14

**Authors:** Kai Xing, Xitong Zhao, Hong Ao, Shaokang Chen, Ting Yang, Zhen Tan, Yuan Wang, Fengxia Zhang, Yibing Liu, HeMin Ni, Yong Guo, Zhuocheng Hou, Chuduan Wang

**Affiliations:** 1 Animal Science and Technology College, Beijing University of Agriculture, Beijing, 102206 China; 2 Key Laboratory of Animal Genetics, Breeding and Reproduction, Ministry of Agriculture, National Engineering laboratory for Animal Breeding, College of Animal Science and Technology, China Agricultural University, Beijing, 100193 China; 3 State Key Laboratory for Animal Nutrition, Key Laboratory for Domestic Animal Genetic Resources and Breeding of the Ministry of Agriculture of China, Institute of Animal Science, Chinese Academy of Agricultural Sciences, Beijing, 100193 China; 4 Beijing General Station of Animal Husbandry, Beijing, 100125 China

**Keywords:** Animal breeding, Gene expression

## Abstract

Fat deposition is very important in pig production, and its mechanism is not clearly understood. MicroRNAs (miRNAs) play critical roles in fat deposition and energy metabolism. In the current study, we investigated the mRNA and miRNA transcriptome in the livers of Landrace pigs with extreme backfat thickness to explore miRNA-mRNA regulatory networks related to lipid deposition and metabolism. A comparative analysis of liver mRNA and miRNA transcriptomes from pigs (four pigs per group) with extreme backfat thickness was performed. We identified differentially expressed genes from RNA-seq data using a Cufflinks pipeline. Seventy-one differentially expressed genes (DEGs), including twenty-eight well annotated on the porcine reference genome genes, were found. The upregulation genes in pigs with higher backfat thickness were mainly involved in fatty acid synthesis, and included fatty acid synthase (FASN), glucokinase (GCK), phosphoglycerate dehydrogenase (PHGDH), and apolipoprotein A4 (APOA4). Cytochrome P450, family 2, subfamily J, polypeptide 34 (CYP2J34) was lower expressed in pigs with high backfat thickness, and is involved in the oxidation of arachidonic acid. Moreover, 13 differentially expressed miRNAs were identified. Seven miRNAs were associated with fatty acid synthesis, lipid metabolism, and adipogenic differentiation. Based on comprehensive analysis of the transcriptome of both mRNAs and miRNAs, an important regulatory network, in which six DEGs could be regulated by differentially expressed miRNAs, was established for fat deposition. The negative correlate in the regulatory network including, miR-545-5p and GRAMD3, miR-338 and FASN, and miR-127, miR-146b, miR-34c, miR-144 and THBS1 indicate that direct suppressive regulation may be involved in lipid deposition and energy metabolism. Based on liver mRNA and miRNA transcriptomes from pigs with extreme backfat thickness, we identified 28 differentially expressed genes and 13 differentially expressed miRNAs, and established an important miRNA-mRNA regulatory network. This study provides new insights into the molecular mechanisms that determine fat deposition in pigs.

## Introduction

MiRNAs are a group of small (approximately 22 nucleotide) non-coding RNAs, which are present in all metazoans and play key roles in diverse biological processes and metabolism^1,2^. In animals, miRNAs regulate gene expression by binding to complementary sequences in untranslated regions of multiple target mRNAs^3^. To date, at least 24,521 miRNAs have been identified in 206 species, including viruses, plants, and animals^4^. However, the biological functions and precise regulatory mechanisms of most miRNAs remain unclear. Paired expression profiling was used to investigate the correlation between miRNA expression patterns and target miRNA, and to identify potential interactions between miRNAs and mRNAs^5–7^.

In pigs, miRNAs have been reported to play important roles in the regulation of fat deposition and energy metabolism. MiR-181a was shown to accelerate the accumulation of lipid droplets and increase the amount of triglycerides by binding to its target, tumor necrosis factor-α (TNF-α)^8^. MiR-302a was found to be a negative regulator of adipocyte differentiation via interaction with the 3ʹ-UTR of peroxisome proliferator activated receptor gamma (PPARγ) mRNA. MiR-27a was also able to accelerate the hydrolysis of triglyceride (TG), and miR-143 was shown to promote TG synthesis during lipid metabolism in porcine adipocytes^9^. In the adipose tissue, miRNAs are differentially expressed in pigs with different genotypes (lean and obese), with miR-9, miR-124a, miR-1a, miR-133a, miR-122, miR-204, and miR-183 all being upregulated in obese pigs. Conversely, the expression of miR-215, miR-135, miR-224, and miR-146b was higher in lean pigs than in obese pigs^10,11^.

The pig (*Sus scrofa*) is an important agricultural animal, and a cost-effective source of meat for human consumption, particularly in China, where pork is the most common meat consumed. Levels of fatness and leanness are very important in pig production because of their ability to affect productive performance, meat quality, and reproductive performance^12,13^. Due to their anatomical, physiological, and genetic similarities to humans, and their high level of fat deposition, pigs represent a suitable model for use in the study of human obesity and energy metabolism^14^. Landrace pigs are a typical lean-type western breed, which have been intensively selected over the past few decades to increase lean meat production and reduce fat deposition^15^. Therefore, fat deposition and metabolism in pigs are always an immediate area of research focus.

The liver is the largest digestive gland and most important metabolic organ. It plays key roles in the regulation of appetite, body weight, and several metabolic processes^16^. In pigs, the main site for *de novo* fatty acid synthesis is the adipose tissue^17^. However, polyunsaturated fatty acid (PUFA) synthesis, *de novo* cholesterol synthesis, and fatty acid oxidation mainly occur in the liver^18,19^. The liver is also critical for glucose metabolism. When blood glucose levels are low, the liver releases glycogen^20^. Liver is suitable tissue to study the molecular mechanisms of fat deposition and metabolism in pigs.

To understand the genes and miRNAs involved in the molecular mechanism of fat deposition in pigs, in the present study we used mRNA and miRNA sequencing to identify differences in liver tissue transcriptomes between pigs with opposing backfat thickness phenotypes. A number of significantly differentially expressed genes and miRNAs were found in the liver tissue from pigs with high and low backfat thickness. Subsequently, the expression patterns and co-expression of differentially expressed mRNA were analysed. Furthermore, we assessed mRNA-miRNA interactions using computational prediction and expression relationship analysis. Finally, integrated transcriptome analysis of mRNA and miRNA in livers was performed in order to identify important networks for differential backfat thickness in pigs.

## Results

### Animal phenotypes

In this study, 132 female landrace pigs, which were on average 186-days-old with an average live body weight of 93.38 kg, were used. Backfat thickness measured 5.76 ± 1.75 mm on average (Table S1). From these data, two groups were selected with low or high backfat thickness respectively. The genealogy information of selected animals is shown in Supplementary Table S2. Backfat thickness of the BFH group (8.88 ± 0.80 mm) was significantly higher than that of the BFL group (3.58 ± 0.39 mm). Additional traits assessed related to fat deposition, such as carcass backfat thickness, kidney fat weight, and intramuscular fat, also exhibited significant differences between the two groups. Individual differences in genetic is the important factor for leading the difference in live fat thickness so significant. There was no difference in body weight between the two groups, indicating that changes in fat deposition were not driven by significant changes in body weight (Table 1).

**Table 1:** Animal performance related to fat deposition in Landrace pigs used in RNA and miRNA sequencing.

	BFH	BFH	P-value
n	4	4	
BW (Kg)	92.65 ± 3.01	89.67 ± 8.95	0.30
BFT(mm)	8.88 ± 0.80	3.58 ± 0.39	1.44E-03
CBFT (mm)	13.30 ± 0.41	7.15 ± 0.05	4.89E-04
LFW (kg)	0.59 ± 0.05	0.33 ± 0.06	8.12E-04
IMF (%)	1.85 ± 0.17	1.37 ± 0.17	3.26E-04

BW – body weight. BFT– backfat thickness (mm) measured at the last 3–4^th^ rib. CBFT- backfat thickness of carcass (mm) at the last 3–4^th^ rib. LFW – leaf fat weight. IMF (%) – percentage of intramuscular fat in the longissimus muscle. p-value as calculated by *t*-test.

### mRNA expression profiles

**Figure 1:**
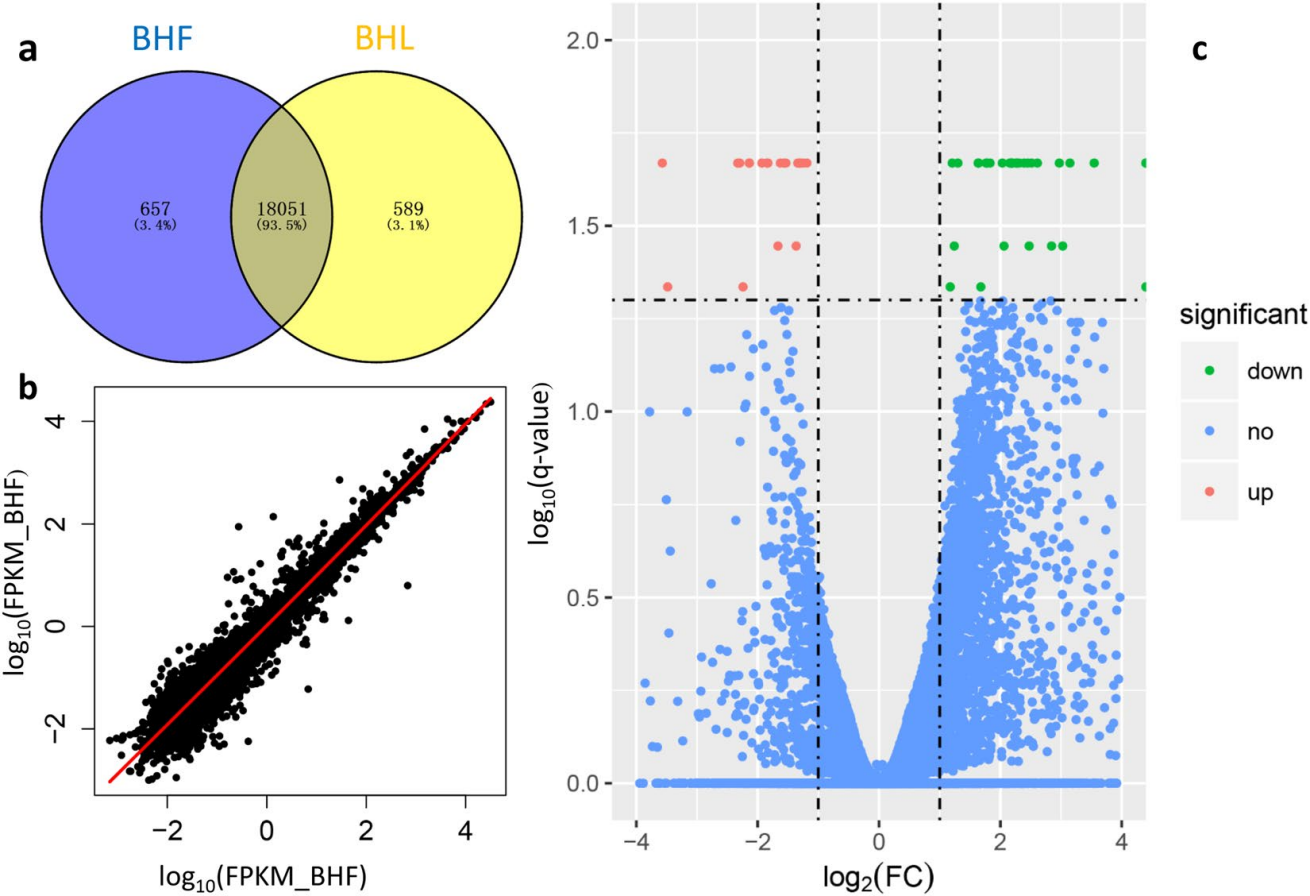
mRNA expression in the livers of pigs with different backfat thickness. (**a**) Venn diagrams show the total number of expressed genes in each individual and both. (**b**) Levels of gene expression in both groups. (**c**) Volcano plot of differentially expressed genes. Significant up-regulation genes are represented as ‘red’ dots and significant downregulation genes are represented as ‘green’ dots on the volcano plot.

In this study, eight cDNA libraries, which included four BFH pigs and four BFL pigs, were sequenced from the liver tissue using Illumina HiSeq. 2000. The deep sequencing data of total RNA have been submitted to National Center for Biotechnology Information (NCBI) Sequence Read Archive (SRA) under Accession no. SRP117778, Bioproject: PRJNA407236. After removing the adaptors and low-quality reads, 23,102,658−27,426,569 clean paired reads, were obtained, with 82.10−86.20% of clean reads being mapped to the porcine reference genome (Table S3). After normalization by FPKM, 19,279 genes were found to be expressed in the liver tissue of all eight pigs. Seventy-one genes were found to be differently expressed between two groups using Cuffdiff (Table S4), with the criteria of at least a 2-fold difference and a q-value of less than 0.05 (Fig. 1). Twenty-eight of the differentially expressed genes were well annotated on the porcine reference genome, including 15 upregulated and 13 downregulated genes for the BFH group (Table 2). Those DEGs not annotated transcript were analyzed through homology with reference genome (GRCh38/hg38). Twenty one of not annotated transcripts were mapped in human reference genome, and eleven genes were found in those locations (Table S5). We also identified 127 DEGs between pigs in half-sib pairs, and 114, 68, and 63 DEGs between pigs in each full-sib pair, respectively. The details of DEGs, including name, expression level and statistical information, are shown in the Supplementary Table S6. No genes were common among the four different pairs (Supplementary Figure S1). However, 29 common genes, such as APOA4, CXCL2, FCN, SAA and so on, were found in more than one pairs. Because of the sample limitations of the DEGs for each pair, we only present DEGs and their functional analyses obtained by treating animals as four biological replicates.

**Table 2:** Twenty-eight well-annotated DEGs in the livers of Landrace pigs with higher and lower fat deposition.

Gene	FPKM (BFH)	FPKM (BFL)	Log_2_(FC)	q_value
ZCCHC8	14.08	4.86	−1.54	0.02
THBS1	2.00	8.17	2.03	0.02
SPP1	13.56	5.36	−1.34	0.02
S1PR3	4.65	1.29	−1.85	0.02
PHGDH	71.21	29.79	−1.26	0.02
MYL9	8.57	19.77	1.21	0.02
LIPG	14.24	5.72	−1.32	0.02
GRAMD3	7.62	2.47	−1.63	0.02
GCK	10.20	2.03	−2.33	0.02
FLNC	0.35	3.12	3.15	0.02
ENSSSCG00000020978	2.12	10.50	2.31	0.02
FGG	1.35	139.30	6.68	0.02
DES	5.02	22.34	2.15	0.02
CYP2J34	1.55	7.47	2.27	0.02
CNN1	2.00	12.22	2.61	0.02
CKS2	7.82	2.64	−1.57	0.02
CD90	12.92	5.27	−1.29	0.02
APOA4	177.44	49.89	−1.83	0.02
ANXA2	131.31	57.54	−1.19	0.02
ADAM8	0.30	8.50	4.82	0.02
ACTG2	1.97	23.05	3.55	0.02
ACTA1	0.00	0.90	inf	0.02
NOR-1	0.10	0.84	3.03	0.04
MYH11	3.00	12.52	2.06	0.04
FASN	148.93	57.80	−1.37	0.04
SEC. 14L2	7.04	2.22	−1.67	0.04
CLEC18A	0.80	4.47	2.47	0.04
ACTA2	17.90	40.36	1.17	0.05

### Functional analysis of differentially expressed genes

**Figure 2:**
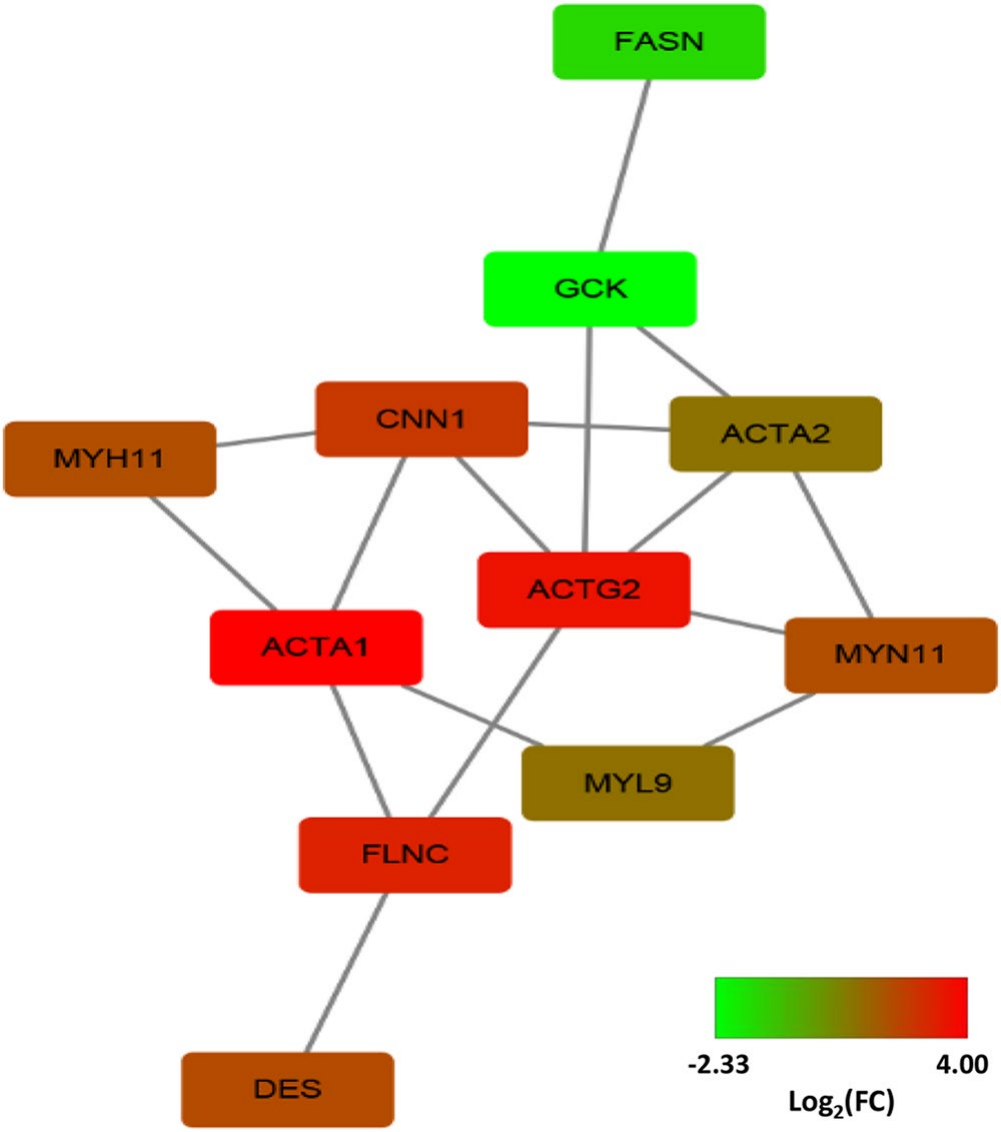
STRING analysis shows that differentially expressed genes are involved in known and predicted protein-protein interactions. STRING is used to analyse differentially expressed genes in the livers of pigs from the BFH and BFL groups. Those up-regulated RNAs were red, and those down-regulated RNAs were green to BFL.

Following conversion to human ortholog ensemble gene IDs, 25 DEGs were annotated in DAVID for pathway and GO analysis (Table S7). Focal adhesion, vascular smooth muscle contraction, and tight junction were identified by KEGG pathway analysis. As little of DEGs, none pathway was found significantly (P < 0.05). For GO functional enrichment analysis, a total of 10 GO terms were enriched (Table 3) in categories related to lipid metabolism (e.g., regulation of lipid transport, regulation of lipoprotein metabolic process, and high-density lipoprotein particle remodelling), and general metabolism (e.g., carbohydrate binding, sugar binding, and regulation of steroid metabolic processes). Gene interaction networks were constructed using STRING online software (Fig. 2). All DEGs were input to the STRING, and those discrete genes in the network was removed.

**Table 3:** Gene ontologies related to metabolism from DEGs in the livers of Landrace pigs.

Genes	Term ID	Term name	P value
**Genes relating to lipid metabolism**			
LIPG, THBS1, APOA4	GO:0032368	regulation of lipid transport	1.17E-03
LIPG, APOA4	GO:0050746	regulation of lipoprotein metabolic process	1.35E-02
LIPG, APOA4	GO:0034375	high-density lipoprotein particle remodelling	2.02E-02
LIPG, APOA4	GO:0034369	plasma lipoprotein particle remodelling	3.35E-02
**Genes related to general metabolism**			
LIPG, CLEC18A, THBS1, GCK	GO:0030246	carbohydrate binding	1.02E-03
CLEC18A, GCK	GO:0005529	sugar binding	9.15E-03
THBS1, SPP1, ACTA1	GO:0045940	regulation of steroid metabolic process	4.17E-02
SEC. 14L2, APOA4	GO:0045940	positive regulation of steroid metabolic process	2.36E-02
LIPG, APOA4	GO:0043691	regulation of cholesterol transport	2.69E-02
LIPG, APOA4	GO:0019218	regulation of sterol transport	3.35E-02

### miRNA expression profiles

**Figure 3:**
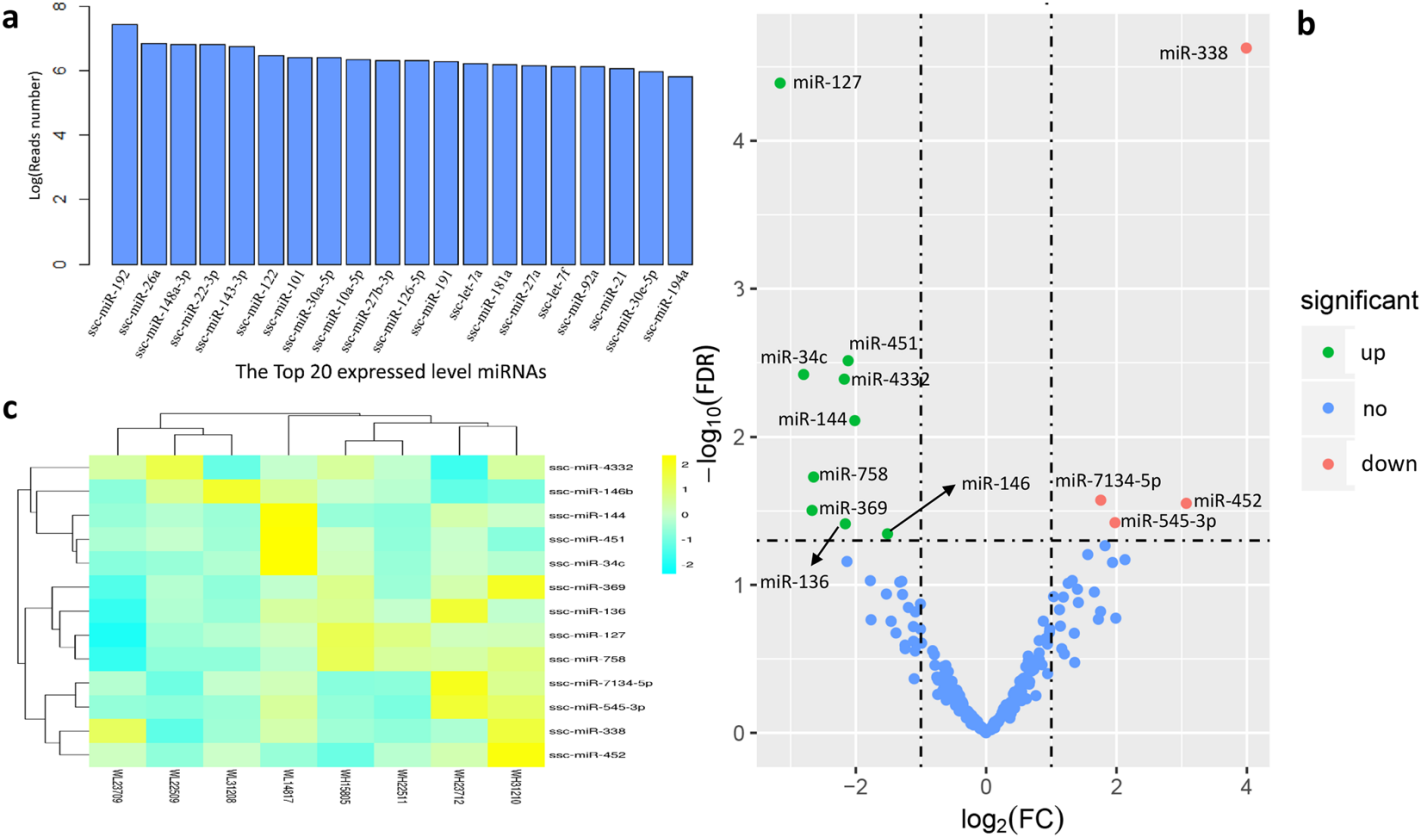
miRNA expression in the livers of pigs with different backfat thickness. (**a**) The expression levels of the most abundant miRNAs. (**b**) Volcano plot of differentially expressed miRNAs. Significant upregulation miRNAs are represented as ‘red’ dots and significant downregulation miRNAs are represented as ‘green’ dots in the volcano plot. (**c**) Hierarchical clustering of differentially expressed miRNAs.

**Figure 4:**
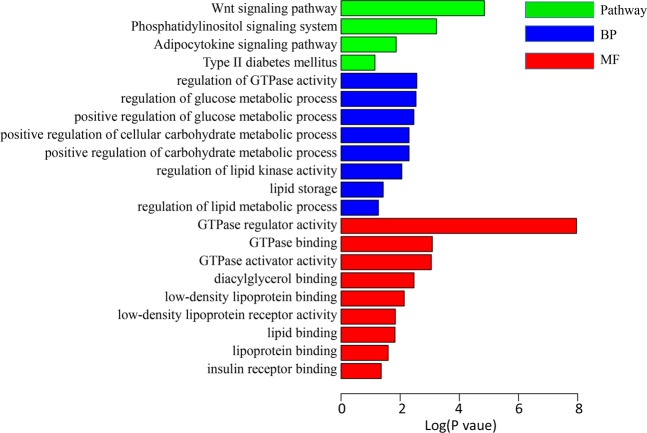
GO terms and KEGG pathways involved in energy metabolism and fat deposition. GO terms and KEGG pathway enrichment analysis for predicted miRNA targets of differentially expressed miRNAs. GO terms and KEGG pathway enrichments were performed by mapping the predicted target genes using DAVID online analysis tool. P < 0.05 was used as a threshold to select significant GO terms and KEGG pathways. −lg(P value) is the negative log10 of the P value. GO, Gene Ontology; BP, biology process; KEGG, Kyoto Encyclopedia of Genes and Genomes.

The deep sequencing data of total RNA have been submitted to NCBI SRA under Accession no. PRJNA407236, Bioproject: PRJNA407256. An average of 19.49 million raw reads were obtained from each individual. After sequencing analysis, 334 miRNAs from all liver samples were identified. In our sequencing data, the miRNAs that were common to the top most abundant miRNAs of all liver samples were: ssc-miR-192, ssc-miR-26a, ssc-miR-148a-3p, ssc-miR-22-3p, ssc-miR-143-3p, ssc-miR-122, ssc-miR-101, ssc-miR-30a-5p, ssc-miR-10a-5p, ssc-miR-27b-3p, ssc-miR-126-5p, ssc-miR-191, ssc-let-7a, ssc-miR-181a, ssc-miR-27a, ssc-let-7f, ssc-miR-92a, ssc-miR-21, ssc-miR-30e-5p, and ssc-miR-194a (Fig. 3). Compared with the BFL group, 13 miRNAs were differentially expressed, including four that were upregulated and nine that were downregulated in the BFH group (Table 4). To further elucidate the functions of the differentially expressed miRNAs, we searched their potential target genes. A total of 3997 putative target genes of 13 DEM were predicted using TARGETSCAN (Table S8). Some putative target genes were repeated. Overall, 2827 target genes were found, and were remained for further analysis. After GO and KEGG pathway analysis, 44 KEGG pathways were identified as being significantly related to genes targeted by differentially expressed miRNAs. Almost all of these pathways are associated with activities that are fundamental to life. However, multiple pathways and GO terms were associated with energy metabolism and fat deposition, such as adipocytokine signalling pathways, regulation of glucose metabolic processes, and lipid binding (Fig. 4).

**Table 4:** Thirteen differentially expressed miRNAs in Landrace pigs with low and high fat deposition.

miRNA	Ref miRNA	Log_2_(FC)	P-value	Mature sequence
ssc-miR-338	hsa-miR-338-3p	3.99	2.36E-05	uccagcaucagugauuuuguug
ssc-miR-127	hsa-miR-127-3p	−3.16	4.07E-05	ucggauccgucugagcuuggcu
ssc-miR-451	hsa-miR-451a	−2.12	3.07E-03	aaaccguuaccauuacugaguu
ssc-miR-34c	hsa-miR-34c-5p	−2.80	3.80E-03	aggcaguguaguuagcugauugc
ssc-miR-4332	—	−2.18	4.09E-03	cacggccgccgccgggcgcc
ssc-miR-144	hsa-miR-144-3p	−2.02	7.79E-03	uacaguauagaugauguac
ssc-miR-758	hsa-miR-758-3p	−2.65	0.02	uuugugaccugguccacuaac
ssc-miR-7134-5p	—	1.76	0.03	auguccgcggguucccuaucc
ssc-miR-452	hsa-miR-452-5p	3.07	0.03	aacuguuugcagaggaaacuga
ssc-miR-369	hsa-miR-369-5p	−2.67	0.03	aauaauacaugguugaucuuu
ssc-miR-545-3p	hsa-miR-545-3p	1.98	0.04	ucaguaaauguuuauuggaug
ssc-miR-136	hsa-miR-136-5p	−2.16	0.04	acuccauuuguuuugaugaugga
ssc-miR-146b	hsa-miR-146b-5p	−1.51	0.05	ugagaacugaauuccauaggc

### Validation of DEGs and DE miRNAs by qPCR

**Figure 5:**
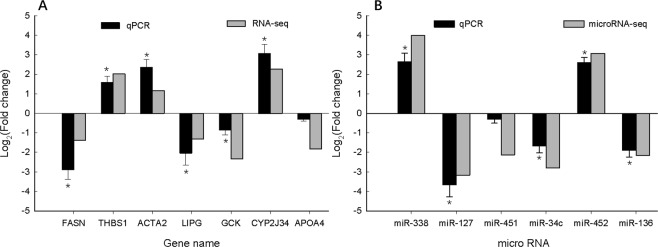
Comparison of transcript expression in terms of fold change as measured by qPCR and NGS. Seven differentially expressed genes (A) and six differentially expressed microRNAs (B) were validated by qPCR. The transcript expression fold changes measured by qPCR and NGS are indicated by dark grey and light grey columns, respectively. Asterisks on the qPCR values indicate significant differences between BFH and BFL at p < 0.05.

We selected seven DEGs (FASN, THBS1, ACTA2, LIPG, GCK, CYP2J34, and APOA4) and six DE miRNAs (miR-338, miR-127, miR-451, miR-34c, miR-452, and miR-136) randomly to validate the accuracy of the RNA-seq and miRNA-seq using qPCR, including the up- and downregulated genes and miRNAs. Furthermore, the fold changes of the seven genes and the six miRNAs in the qPCR and in the RNA-seq or miRNA-seq showed the same trends (Fig. 5). Most of them were significant differently expressed between two groups (p < 0.05). The correlation between RNA-seq and qPCR is 0.84 (p < 0.05), and the correlation between microRNA-seq and qPCR is 0.91 (p < 0.05). Those results indicated that the DEGs and DE miRNAs identified with NGS were reliable and efficient.

### Construction of the miRNA/mRNA network

**Figure 6:**
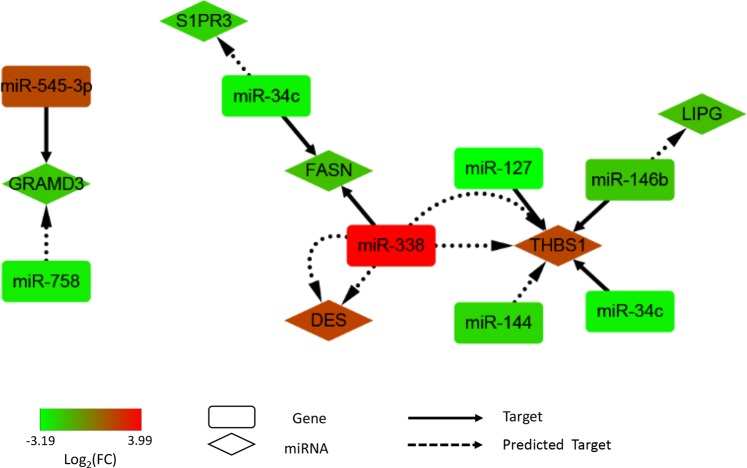
Key network of genes and miRNAs differentially expressed in the livers between pigs with extreme backfat thickness. The network diagram was generated using Cytoscape. Those up-regulated RNAs or miRNAs were red, and those down-regulated RNAs or miRNAs were green to BFL.

To further analyse the relationship between the node mRNAs and non-coding miRNAs, a potential network of DEGs and differentially expressed miRNAs interacting in pig liver that might affect fat deposition was constructed (Fig. 6). The node mRNAs and non-coding miRNAs indicate that those mRNAs and non-coding miRNAs have potential regulatory relationships. In the miRNA/mRNA network, six DEGs, including GRAM domain containing 3 (GRAMD3), FASN, desmin (DES), thrombospondin 1 (THBS1) and lipase G, endothelial type (LIPG), could be regulated by differentially expressed miRNAs. MiR-338 targeted FASN, and their expression level shown negative relation (correlation coefficient = −0.52). The correlation coefficient between miR-127, miR-146b, miR-144, miR-34c and their potential targeted gene THBS1 were −0.90, −0.27, −0.90 and −0,94, respectively. GRAMD3 could be negative regulated by miR-545-3p, and their correlation coefficient is −0.83.

## Discussion

In this study, three full-sib pairs of pigs and one half-sib pair of individuals with extreme backfat thickness phenotypes were used. Landrace pigs is an lean-type pigs, and have an low level of fat deposition as an whole. But phenotypic variation of fatty traits such as backfat thickness still was found in this population. There were no differences in the body weight of pigs from different groups; however, significant differences in traits related to fat deposition such as carcass backfat thickness, kidney fat weight, and intramuscular fat, were observed. This suggested that the significant changes in fat deposition were not mediated by changes in body weight. To our knowledge, this is the first study to perform parallel comparisons between mRNA and miRNA transcriptomes in the liver in order to investigate the function of mRNAs, the roles of miRNAs, and their targets in lipid deposition and metabolism. In contrast with previous studies^21–23^, the use of more full-sib pairs may reduce the noise associated with differences in the digenetic background and the generation of false-positive results. Comparing the result of each pair, there were more DEGs in half-sib pair than that in any full-sib pair. Thus, selected full-sib pigs represent a better sampling strategy to assess differences in gene expression.

After removal of low-quality reads, between 82.1% and 86.2% of the obtained sequence reads, each sample was mapped to the porcine reference genome. These values were higher than those reported in previous studies investigating the porcine liver transcriptome: 72.63^24^, 64.46^25^, 71.42–77.75^26^ and 78.29–79.60%^27^. This represents a good result for RNA-seq data analysis. The total number of genes expressed in the liver was similar between groups (BFH = 18,708, BFL = 18,640), and 18,051 common genes were expressed in both groups. This suggests that most of the liver transcriptome is conserved between animals of the two groups. Only 28 DEGs were identified in the livers of pigs with significantly different backfat thickness. The number of DEGs was lower than that reported in several studies that performed porcine liver transcriptomic analyses using RNA-seq to identify genes related to fat deposition: 169^23^, 1,551^25^ and 92^28^. Compared to previous studies, increasing the number of samples and ensuring adequate control of the genetic backgrounds could reduce false-positive results. However, 11 DEGs (actin, alpha 2, smooth muscle, aorta [ACTA2], APOA4, CYP2J34, fibrinogen gamma chain [FGG], GRAMD3, myosin light chain 9 [MYL9], DES, FASN, actin, alpha 1, smooth muscle, aorta [ACTA1], filamin C [FLNC], and NADP-dependent oxidoreductases 1[NOR-1]) were included in the list of DEGs found in the above-mentioned study. Therefore, those 11 genes may play an important role in the regulation of fat deposition in the liver. Biological functions of the DEGs are candidate functions to explain the variation in fat deposition between analysed animals. Most of the significant gene ontology (GO) terms belong to lipid metabolism and general metabolism. Due to the low number of DEGs, nominal p-values (P < 0.05) rather than corrected p value (P < 0.05) is the threshold value as significance level in GO enrichment analysis. Several GO terms, such as regulation of lipid transport, regulation of lipoprotein metabolic process, and carbohydrate binding, were identified as significant categories. APOA4 and LIPG are involved in processes related to several GO terms, such as those related to lipid metabolism and general metabolism^29–31^, and were up-regulated in the BFH group in this study. This suggests that fat content in the liver is subjected to constant regulation by negative feedback. GCK, which was more highly expressed in the BFH groups, is involved in the first step of glucose metabolism, and controls gluconeogenesis and glycogen synthesis in the liver^32^. This shows that the livers of pigs with high backfat thickness possess a high level of fatty acid synthesis. Hence, differences in the rates of *de novo* fatty acid synthesis, and lipid metabolism in the liver between leaner pigs, compared with fatter pigs, are attributable to genetic factors resulting in differential fat deposition.

In this study, liver microRNAomes were compared between BFH and BFL, and 13 differentially expressed miRNAs were identified. Several differentially expressed miRNAs were considered to play regulatory roles in fatty acid synthesis, lipid metabolism, and adipogenic differentiation, and similar results have been reported previously. For example, miR-338 was specifically detected in the adipose tissue of leaner pigs^33^, and was also found to be significantly down-regulated in the adipose tissue of obese pregnant women^34^. MiRNAs differentially expressed in Large White pigs (lean type pig) and Meishan pigs (Chinese indigenous fatty pig) have been identified through a high-throughput Solexa sequencing approach. The results showed that the expression of miR-136 in Meishan pigs was significantly higher than that in Large White pigs^10^. Expression of miR-144 has been reported to cause diseases related to lipid metabolism^35,36^. Abundance expression of miR-4332 was shown to be higher in lean pigs (Landrace and Yorkshire) than in obese pigs ((Diannan Small-ear pig and Tibetan pig)^37^. These data are consistent with the results of the present study.

The miRNA-mRNA regulatory networks identified in this study provide a comprehensive profile that can help to delineate the mechanisms of fat deposition. MiRNA acts a negative regulator of gene expression in fat formation^38^. GRAMD3 has been reported as a candidate gene associated with ectopic fat^39^, and is a potential target gene of miR-545-3p. GRAMD3 and miR-545-3p display opposing patterns of expression in the two groups. Highly negative correlation between GRAMD3 and miR-545-3p (−0.83) means miR-545-3p may regulate fat deposition through targeted GRAMD3. FASN is a key lipogenic enzyme and the rate-limiting step in *de novo* fatty acid synthesis^40^. Consistent with previous reports^41^, FASN expression was higher in the fatter pigs in our study. MiR-338 was upregulated in the liver of animals in the BFL group, and is predicted to target FASN. This implies the expression of FASN could be negatively regulated by miR-338. THBS1 represents the hub molecules in the network, plays a causal role in the pathogenesis of insulin resistance and adipose tissue inflammation, and serves as a biological marker of obesity and the metabolic syndrome^42^. MiR-127, miR-146b, miR-34c and miR-144 have been predicted to regulate the level of THBS1 expression. In our study, miR-127, miR-146b, miR-34c and miR-144 were upregulation in the liver of animals in the BFH group, whereas THBS1 was downregulated in animals of this group. Those potential negative regulation between miR-127, miR-34c, miR-144 and THBS1 may play important roles in fat deposition. As it represents the target off our identified differentially expressed miRNAs, THBS1 represents a new candidate gene influencing backfat thickness in pigs. However, the expression level of proteins and the specific functions of these crucial genes and miRNAs have not been studied in depth, so further studies are needed to understand fat deposition in pigs.

## Conclusion

In the present study, we identified 28 genes and 13 miRNAs that were differentially expressed in the livers of Landrace pigs with extreme and divergent backfat thickness in this population. Genes functionally related to fatty acid synthesis, glucose metabolism, and fatty acid degradation were found to be differentially expressed. Additionally, a few miRNAs that target genes involved in fatty acid synthesis, lipid metabolism, and adipogenic differentiation were also found to be differentially expressed. Based on the comprehensive transcriptome analysis of both mRNAs and miRNAs, we conclude that the levels of fatty acid synthesis and degradation in liver are due to the degree of fat deposition. Adipogenic differentiation in the liver regulated by miRNAs could influence body fat of pigs. Overall, we modelled the miRNA-mRNA regulatory network related to fatty acid synthesis and lipid transport using data on differentially expressed miRNAs and mRNAs from Landrace pigs with extreme and divergent backfat thickness. The negative correlate including, miR-545-5p and GRAMD3, miR-338 and FASN, and miR-127, miR-146b, miR-34c, miR-144 and THBS1 indicate a possible direct suppressive regulation between miRNAs and their target mRNAs. The overall results of this study facilitate our understanding of the molecular mechanisms regulating fat deposition in pigs and provide an insight into a regulatory role for miRNAs in energy metabolism.

## Materials and Methods

### Animals and phenotypes

A landrace female pig resource population was housed in the Tianjin Ninghe Primary Pig Breeding Farm (Ninghe, China) under consistent and standard environmental conditions. Animals were fed three times a day and had free access to water. A total of 132 individuals were measured at the last 3rd and 4th ribs using real-time B-mode ultrasonography (HS1500 convex scanner, Honda Electronics, Toyohashi, Japan) to determine live backfat thickness. Age, body weight, and pedigree information were also available for the resource population. Selection methods and standards included two criteria: live backfat thickness in pigs from the high backfat thickness group (BFH) was at least twice that of pigs from the low backfat thickness group (BFL), and the pairs of pigs with divergent backfat thickness, which were full-sibs, were the first choice for selection. Four pairs of pigs with divergent backfat thickness phenotypes, three of which were full-sibs, were selected, slaughtered, and used for the collection of livers. Each pairs were traced three generations in pedigree, and had no relationship between ancestors.

After being stunned with a captive bolt and exsanguinated, four pairs of selected pigs were slaughtered in a commercial abattoir (Beijing Huadu Sunshine Food Co., Ltd., Beijing, China). All chosen pigs were stunned with a captive bolt, exsanguinated and slaughtered in commercial abattoir called Beijing Huadu Sunshine Food co., LTD. The chosen pigs were slaughtered according to guidelines of operating procedures of pig-slaughtering (GB/T 17236–2008), which was promulgated by General Administration of Quality Supervision, Inspection and Quarantine of the People’s Republic of China (AQSIQ) and Standardization Administration of the People’s Republic of China (SAC). All efforts to minimize animal suffering were made during the study. The whole procedure for collection of the tissue samples of all animals was by our researchers. This study was approved specifically by the Animal Welfare Committee of China Agricultural University (Permit number: DK996). All protocols and procedures involving animals were performed in accordance with the Regulations for the Administration of Affairs Concerning Experimental Animals. The liver tissue was collected aseptically and stored in liquid nitrogen immediately after slaughter until required for RNA isolation. The backfat thickness of carcasses at the last 3–4^th^ ribs and leaf fat weight were measured on slaughtered pigs. The percentage of intramuscular fat in the longissimus muscle was quantified by a near infrared technique Foodscan (Foss CO. Ltd., Sweden) and the value was expressed as the weight percentage of wet muscle tissue. The significance of the difference in fatness traits between the two groups was assessed using t-test by R^43^.

### RNA isolation, library preparation, and sequencing

Total RNA was isolated from eight livers using TRIzol Reagent (Invitrogen, CA, USA) according to the manufacturer’s recommendations. The concentration and quality of RNA were validated by measuring the absorbance at 260/280 nm (A260/A280) using Smart Spec Plus (Bio-Rad, USA). The RNA integrity was further verified by 1.5% agarose gel electrophoresis and an Agilent 2100 Bioanalyzer (Agilent Technologies, CA, USA). For each of eight samples, 10 μg of RNA was used for RNA-seq library preparation using the TruSeq® Stranded Total RNA Sample Preparation Kit (Illumina®). The procedures and standards were performed according to the kit’s instructions. After purification and enrichment, all libraries were sequenced using an Illumina HiSeq. 2000 (Illumina, CA, USA). In this experiment, two lanes were used in the Illumina HiSeq. 2000 for mRNA and miRNA sequencing, respectively.

### mRNA sequencing data statistical analysis

Paired-end (PE) libraries were prepared according to the Illumina paired-end library preparation protocol (Illumina, San Diego, CA), and were sequenced on an Illumina Hiseq. 2000 sequencing system to generate 2 × 90 PE reads. High-quality reads of PE were obtained for each sample. Adaptor sequences, reads with more than 10% unknown sequences, “N”, and low-quality sequences (the percentage of low-quality bases with a threshold quality score <20) were removed. The obtained sequence reads were quality-checked by FastQC^44^. Clean reads were aligned to the porcine reference genome sequence (Sscrofa11.1) using TopHat v2.0.1 software^45^. The porcine genome (Sscrofa11.1) was obtained from Ensembl database. Reads aligned to the reference genome were assembled by Cufflinks software^46^. Cuffdiff, a part of the Cufflinks package, was used to identify differentially expressed genes (DEGs) and transcripts from two groups of pigs with opposing backfat thickness performance^47^. The threshold value for selection of differentially expressed genes is q-value ≤ 0.05 and fold change (FC) ≥ 2 or ≤ 0.5, as |Log_2_ FC| ≥ 1. Moreover, DEGs between pigs in each pair were also analyzed using the same analysis pipeline.

### miRNA sequencing and statistical analysis

Eight small RNA libraries were prepared using the TruSeq® Small RNA Sample Prep Kit (Illumina®). All of the procedures and standards were performed as described in the instructions supplied with the kit. After quality control, eight libraries were sequenced on a HiSeq. 2000 platform (Illumina) and 50-bp single-end reads were obtained. Before reads were analysed, the adapters were removed and low-quality reads were discarded using the Fastx-toolkit for quality control^48^. The CAP-miRSeq pipeline was used for miRNA analysis^49^. MiRNA data were converted from fatsq to fasta format using awk in the Linux system. MirDeep2 was used to analyse miRNA sequencing data^50^. The miRNA reference was downloaded from the miRBase database^4^. The expression of microRNAs in different libraries was normalised by Trimmed Mean of M-values (TMM)^51^, and differentially expressed miRNAs (P-value ≤ 0.05, and FC ≥ 2 or ≤ 0.5, as |Log2 FC| ≥ 1) were identified using the edgeR package^52^.

### Quantitative real-time PCR (qPCR) of mRNA and miRNA

Reverse transcription quantitative real-time PCR were used to confirm the sequencing data by Light Cycler® 480 Real-Time PCR System (Roche, USA). Seven differentially expressed genes and six differentially microRNAs were selected randomly. Total RNA was isolated from four pairs pigs and converted into cDNA using the Revert Aid™ First Strand cDNA Synthesis Kit (Thermo Fisher Scientific Inc, USA). The reaction volume of 20 μL was used in the qPCR reactions according to the manufacturer’s protocol. The primers used are shown in Table S9. Triplicate qPCRs were performed for each cDNA and the average Ct was used for further analysis. The 2^−ΔΔCt^ method was used to determine the relative mRNA abundance^53^. To confirm changes in miRNA levels between BJ and HL groups, parallel qPCRs were also used to quantify relevant miRNAs. Six differential expressed microRNAs were selected for qPCR analysis. Chicken U6 was chosen as an endogenous control to correct for analytical variations. This experiment was performed by Beijing SinoGene Scientific Co., Ltd.

### Functional analysis

Gene IDs were converted to homologous human Ensembl Gene Ids using bioDBnet^54^ (https://biodbnet-abcc.ncifcrf.gov/) and subjected to functional analysis. The Database for Annotation, Visualization and Integrated Discovery (DAVID)^55^ bioinformatics resources (http://david.abcc.ncifcrf.gov/) were used for Kyoto Encyclopedia of Genes and Genomes (KEGG) pathway and Gene Ontology (GO) enrichment analysis of differentially expressed genes using nominal p-values (P < 0.05) including molecular functions (MFs), biological processes (BPs), and cellular components (CCs). An interaction network of DEGs was structured using the Search Tool for the Retrieval of Interacting Genes (STRING) database^56^ (http://string-db.org/). To explore the potential functions of differentially expressed miRNAs, target genes were predicted using the miRBase database^4^ (http://www.microrna.org/) based on homologous human genes, since the porcine data were not available in the current versions^57^. Target genes validated by differentially expressed miRNAs were identified by DIANA-TarBase^58^. The DEGs, those that existed in the cluster of predicted or validated targets, were considered to be potential porcine target genes. The network diagram was generated using Cytoscape^59^. The Pearson’s correlation analysis between target genes and potential miRNA was performed using the SPSS 13.0 for Windows. Their correlation coefficient was calculated by their expressed level in the high-throughput sequencing data.

## Supplementary information

Supplementary Informations

Dataset

## Data Availability

All the basic data series were submitted to NCBI SRA with accession number SRP117778 (BioProject: PRJNA407236) and SRP117771 (BioProject: PRJNA407256).
