# Supplementary material for: Transcriptome analysis of miRNA and mRNA in the livers of pigs with highly diverged backfat thickness
Source: Sci Rep. 2019 Nov 14;9:16740. doi: 10.1038/s41598-019-53377-x (PMC6856533; doi:10.1038/s41598-019-53377-x)

# Transcriptome analysis of miRNA and mRNA in the livers of pigs with highly diverged backfat thickness

Kai Xing[1,2], Xitong Zhao[2], Hong Ao[3], Shaokang Chen[4], Ting Yang[2], Zhen Tan[2], Yuan Wang[2], Fengxia Zhang[2], Yibing Liu[2], HeMin Ni[1], Yong Guo[1*], Zhuocheng Hou[2*]and Chuduan Wang[2*]

[1] Animal Science and Technology College, Beijing University of Agriculture, Beijing, 102206, China

[2]Key Laboratory of Animal Genetics, Breeding and Reproduction, Ministry of Agriculture, National Engineering laboratory for Animal Breeding, College of Animal Science and Technology, China Agricultural University, Beijing 100193, China

[3]State Key Laboratory for Animal Nutrition, Key Laboratory for Domestic Animal Genetic Resources and Breeding of the Ministry of Agriculture of China, Institute of Animal Science, Chinese Academy of Agricultural Sciences, Beijing 100193, China

[4]Beijing General Station of Animal Husbandry, Beijing 100125, China

*Correspondence:

YG: y63guo@126.com

ZH: zchou@cau.edu.cn

CW: cdwang@cau.edu.cn

*Corresponding author

Table S2. The genealogy information of selected animals.

| Sample ID | Boar ID | Sow ID | Litter ID |
|---|---|---|---|
| 14817 | LLNHPF708007239 | LLNHPF708006338 | 148 |
| 15805 | LLNHPF708007239 | LLNHPF710007876 | 158 |
| 22509 | LLNHPF111011101 | LLNHPF709002652 | 225 |
| 22511 | LLNHPF111011101 | LLNHPF709002652 | 225 |
| 23709 | LLNHPF710006737 | LLNHPF108001825 | 237 |
| 23712 | LLNHPF710006737 | LLNHPF108001825 | 237 |
| 31208 | LLNHPF710001599 | LLNHPF109005690 | 312 |
| 31210 | LLNHPF710001599 | LLNHPF109005690 | 312 |

14817 and 15805 are half-sib pair; 22509 and 22511 are full-sib pair; 23709 and 23712 are full-sib; 31208 and 31210 are full-sib.

Table S3. The mapping rate of each sample

| Sample ID | 14817 | 15805 | 23712 | 23709 | 31210 | 31208 | 22509 | 22511 |
|---|---|---|---|---|---|---|---|---|
| Raw reads number | 27734508 | 25868236 | 29167050 | 30577066 | 28546699 | 29654984 | 27614544 | 27895944 |
| Clean reads number | 24962054 | 23102658 | 26276375 | 27426569 | 25629568 | 26740904 | 24871531 | 25008304 |
| Left reads mapped | 20642000 | 19036539 | 22813393 | 23786251 | 21378352 | 23077371 | 20658022 | 20874850 |
| Right reads mapped | 20435152 | 18886352 | 22138138 | 23495630 | 22181574 | 22834309 | 20282848 | 20602969 |
| Reads mapped rate | 82.3% | 82.1% | 85.5% | 86.2% | 85.0% | 85.8% | 82.3% | 82.9% |

Table S5. DEGs through homology with human reference genome

| Gene_ID | locus | Gene stable ID | Gene name | chromosome | Gene start (bp) | Gene end (bp) |
|---|---|---|---|---|---|---|
| XLOC_023053 | 4:118767853-118768431 | ENSG00000171385 | KCND3 | 1 | 111770662 | 111989155 |
| XLOC_022825 | 4:96310880-96317115 | ENSG00000198929 | NOS1AP | 1 | 162069774 | 162370475 |
| XLOC_009845 | 14:60362808-60377442 | ENSG00000238005 | AL391832.2 | 1 | 234957342 | 234970062 |
| XLOC_029886 | 7:96763348-96763667 | ENSG00000171723 | GPHN | 14 | 66507407 | 67181803 |
| XLOC_001134 | 1:310241039-310241573 | ENSG00000122490 | PQLC1 | 18 | 79902420 | 79951664 |
| XLOC_021066 | 3:124268443-124292801 | ENSG00000231200 | LINC01830 | 2 | 21933336 | 22531105 |
| XLOC_021066 | 3:124268443-124292801 | ENSG00000228999 | AC068490.1 | 2 | 21942178 | 21965680 |
| XLOC_009025 | 13:108862113-108865348 | ENSG00000163590 | PPM1L | 3 | 160755602 | 161078907 |
| XLOC_033496 | 9:102509899-102510297 | ENSG00000085563 | ABCB1 | 7 | 87503633 | 87713323 |
| XLOC_015726 | 18:34021752-34022173 | ENSG00000128573 | FOXP2 | 7 | 114086327 | 114693772 |
| XLOC_015562 | 18:19001647-19008040 | ENSG00000226380 | AC016831.1 | 7 | 130876809 | 130913310 |

Table S7. The list of DEGs annotated in DAVID

| Gene Symbol | Homolog - Human Ens Gene ID |
|---|---|
| ZCCHC8 | ENSG00000033030 |
| THBS1 | ENSG00000137801 |
| SPP1 | ENSG00000118785 |
| S1PR3 | ENSG00000213694 |
| PHGDH | ENSG00000092621 |
| MYL9 | ENSG00000101335 |
| LIPG | ENSG00000101670 |
| GRAMD3 | ENSG00000155324 |
| GCK | ENSG00000106633 |
| FLNC | ENSG00000128591 |
| FGG | ENSG00000171557 |
| DES | ENSG00000175084 |
| CYP2J34 | ENSG00000134716 |
| CNN1 | ENSG00000130176 |
| CKS2 | ENSG00000123975 |
| APOA4 | ENSG00000110244 |
| ANXA2 | ENSG00000182718 |
| ADAM8 | ENSG00000151651 |
| ACTG2 | ENSG00000163017 |
| ACTA1 | ENSG00000143632 |
| MYH11 | ENSG00000133392 |
| FASN | ENSG00000169710 |
| SEC14L2 | ENSG00000100003 |
| CLEC18A | ENSG00000140839 |
| ACTA2 | ENSG00000107796 |

Table S9. Primers used in qPCR for eight genes

| Gene name | forward primer (5'-3') | reverse primer (5'-3') |
|---|---|---|
| FASN | GGGCCCAGCATCACCATAGACA | GTTCGTGCCCGCATTGAGGAT |
| THBS1 | ACCACGACAAGGATGGCAAG | TCGGCGGAAATCAGTCTCAC |
| ACTA2 | AGAACACGGCATCATCACCAACTG | CACCGCCTGAATAGCCACATACAT |
| LIPG | TGCAACAGCCAAAACCTTCT | TGTCCCACTTTCCTCGTGTT |
| GCK | AGCCCAGTGCTGACCCTCTA | GGATTTCCTGTGCCTTCTTTA |
| CYP2J34 | CCCTCANTTCAAGATCAACA | GCAGATGAGGTTTTCTTCAT |
| APOA4 | GATGAAGAAGCAGGCAGAGG | TGTTGAAGGTCTCCCCGTAG |
| GAPDH | GGTCACCAGGGCTGCTTTTA | CCTTGACTGTGCCGTGGAAC |

Figure S1. the VN of DEGs between pigs in pair

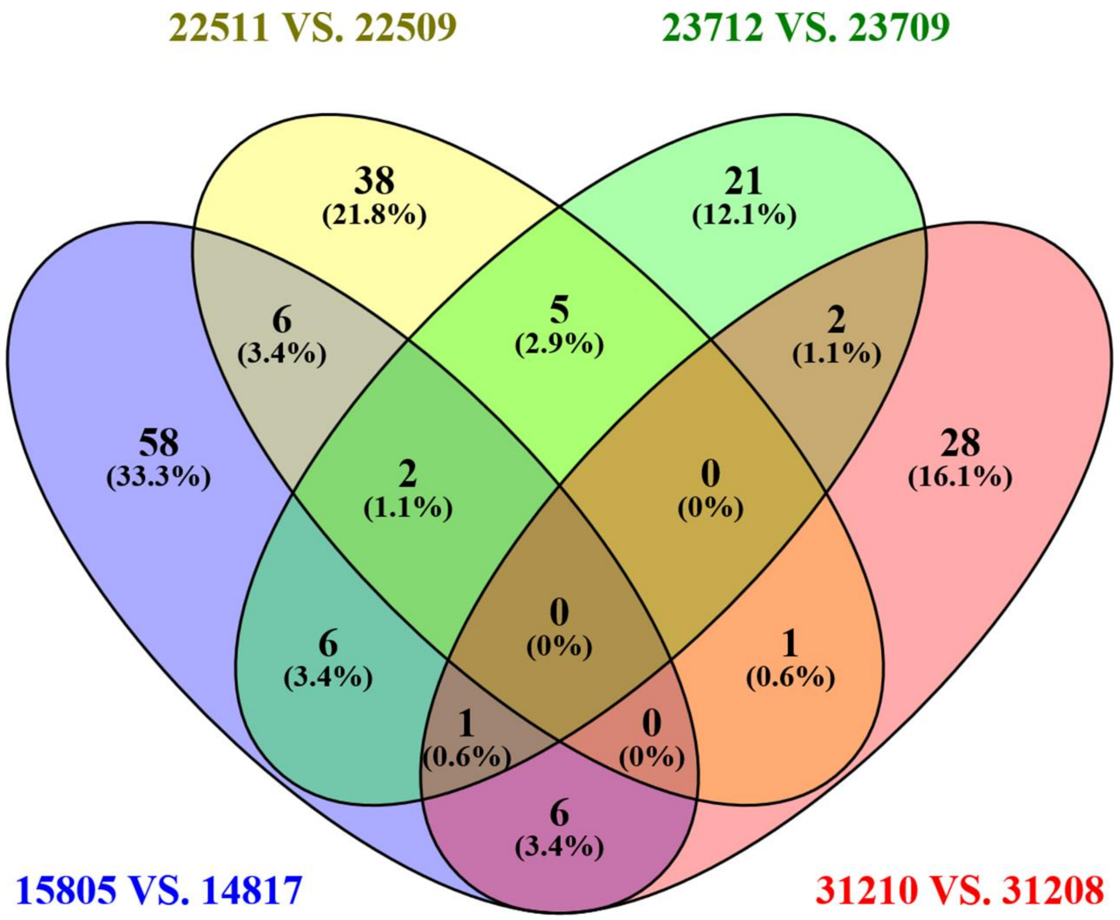

Supplement: Supplementary file 1 — Supplementary Informations [file 41598_2019_53377_MOESM1_ESM.pdf]
